# MRI-Derived Tumour-to-Breast Volume Is Associated with the Extent of Breast Surgery

**DOI:** 10.3390/diagnostics11020204

**Published:** 2021-01-30

**Authors:** Andrea Cozzi, Simone Schiaffino, Gianmarco Della Pepa, Serena Carriero, Veronica Magni, Diana Spinelli, Luca A. Carbonaro, Francesco Sardanelli

**Affiliations:** 1 Department of Biomedical Sciences for Health, Università degli Studi di Milano, Via Luigi Mangiagalli 31, 20133 Milano, Italy; andrea.cozzi1@unimi.it (A.C.); veronica.magni@unimi.it (V.M.); francesco.sardanelli@unimi.it (F.S.); 2 Unit of Radiology, IRCCS Policlinico San Donato, Via Rodolfo Morandi 30, 20097 San Donato Milanese, Italy; luca.carbonaro@grupposandonato.it; 3 Postgraduate School in Radiodiagnostics, Università degli Studi di Milano, Via Festa del Perdono 7, 20122 Milano, Italy; gianmarco.dellapepa@unimi.it (G.D.P.); serena.carriero@unimi.it (S.C.); diana.spinelli@unimi.it (D.S.)

**Keywords:** breast-conserving surgery, breast neoplasms, magnetic resonance imaging, mastectomy, tumour-to-breast volume ratio

## Abstract

The tumour-to-breast volume ratio (TBVR) is a metric that may help surgical decision making. In this retrospective Ethics-Committee–approved study, we assessed the correlation between magnetic resonance imaging (MRI)-derived TBVR and the performed surgery. The TBVR was obtained using a fully manual method for the segmentation of the tumour volume (TV) and a growing region semiautomatic method for the segmentation of the whole breast volume (WBV). Two specifically-trained residents (R1 and R2) independently segmented T1-weighted datasets of 51 cancer cases in 51 patients (median age 57 years). The intraobserver and interobserver TBVR reproducibility were calculated. Mann-Whitney *U*, Spearman correlations, and Bland-Altman statistics were used. Breast-conserving surgery (BCS) was performed in 31/51 cases (61%); mastectomy was performed in 20/51 cases (39%). The median TBVR was 2.08‰ (interquartile range 0.70–9.13‰) for Reader 1, and 2.28‰ (interquartile range 0.71–9.61‰) for Reader 2, with an 84% inter-reader reproducibility. The median segmentation times were 54 s for the WBV and 141 s for the TV. Significantly-lower TBVR values were observed in the breast-conserving surgery group (median 1.14‰, interquartile range 0.49–2.55‰) than in the mastectomy group (median 10.52‰, interquartile range 2.42–14.73‰) for both readers (*p* < 0.001). Large scale prospective studies are needed in order to validate MRI-derived TBVR as a predictor of the type of breast surgery.

## 1. Introduction

Over the years, improvements in breast cancer care have been driven by advances in prevention and treatment [1,2]. The management of early-stage breast cancer reached a turning point when Halsted mastectomy was gradually replaced by breast-conserving surgery, followed by whole-breast radiation therapy (i.e., breast-conserving treatment), as a consequence of large randomized trials conducted in the 1970s and 1980s [3,4,5,6]. These studies showed no difference both in the disease-free and overall survival of patients who underwent conserving treatment compared to radical mastectomy [1], while improving patients’ satisfaction and quality of life [7,8] by obtaining complete tumour removal as well as favourable cosmetic results [9].

Recognizing the benefits of breast-conserving treatment [7,8,9], the European Society of Breast Cancer Specialists set a minimum 70% rate of patients diagnosed with invasive breast cancer who subsequently undergo conserving surgery as a quality indicator [10]. However, conserving surgery is hampered by difficulties related to large tumour size, small breast volume, multicentric or extended multifocal cancers, and tumour locations that are unsuitable for conserving surgery, all potentially leading to poor cosmetic results or positive resection margins needing re-excision [11,12].

In clinical practice, a preoperative standardized assessment of the expected cosmetic outcome after conserving surgery is lacking. A large tumour volume relative to the total breast volume and tumour location is known to be a relevant predictive factor of poor cosmetic results [13,14]. In this context, Vos et al. [15] have recently defined a preoperative prediction model of cosmetic results based on tumour-to-breast volume ratio (TBVR) and tumour location. However, TBVR measurement has yet to enter routine use, nor has it been consistently associated with a specific imaging modality, even though automated breast ultrasound and breast magnetic resonance imaging (MRI) are chiefly considered for such assessment as a consequence of being three-dimensional imaging methods [16,17,18,19,20,21,22,23,24]. Breast MRI, which is frequently used to guide surgical planning [25], has been associated more commonly than automated breast ultrasound with an accurate assessment of tumour size and of overall breast volume [26]. However, several issues, such as the time-consuming and operator-dependent nature of manual image segmentation, need to be carefully addressed in order to establish the role of MRI-derived TBVR in the preoperative setting.

In this scenario, we aimed to evaluate the interobserver reproducibility of TBVR measured by MRI using a fully manual method for tumour volume segmentation and a semiautomatic segmentation method for whole breast volume segmentation, and to investigate the correlation between the MRI-derived tumour volume and final pathology, as well as between TBVR and surgical choices.

## 2. Materials and Methods

### 2.1. Study Protocol

The approval for this monocentric retrospective study was obtained by the Ethics Committee of IRCCS Ospedale San Raffaele, Milano; protocol code SenoRetro; approved on 9 November 2017 and amended on 18 July 2019.

### 2.2. Patient Cohort

We conducted a search on our institutional database for all of the percutaneous image-guided biopsies performed between 1 January 2013 and 31 May 2019. We then selected all of the patients who had a histologically-confirmed malignant lesion—visible in the breast MRI as a mass lesion—and available results of the final pathology on surgical specimen. Women with incomplete or technically-suboptimal breast MRI examinations, breast implants, associated non-mass lesions on MRI scans, multicentric tumours, and who underwent neoadjuvant chemotherapy were excluded from analysis. The demographics, imaging, and pathology data were subsequently retrieved for each included patient, along with the patient-specific surgical choice (mastectomy versus conserving surgery).

### 2.3. Image Acquisition

All of the MRI examinations were performed with the patient placed in a prone position on a 1.5-T unit (Sonata Symphony class, Siemens Healthineers, Erlangen, Germany) with gradients up to 40-mT/m, using bilateral four-element dedicated phased-array coils.

The image acquisition started with a triplane scout view, followed by a series of bilateral axial sequences: (A) T2-weighted short-tau inversion recovery (inversion time 150 ms, flip angle 150°); (B) fat-saturated echo-planar diffusion-weighted imaging (*b*-values 0 and 750 s/mm^2^); (C) dynamic contrast-enhanced T1-weighted three-dimensional fast low-angle shot spoiled gradient-echo, acquired once before and four times after the intravenous injection of a 0.1 mmol/kg dose of a gadolinium-based contrast agent (gadobenate dimeglumine, Bracco Imaging, Milan, Italy, or gadobutrol, Bayer Healthcare, Berlin Germany), with a temporal resolution of 119 s. Other details on the acquisition parameters are listed in Table 1.

### 2.4. Image Analysis and Processing

**Figure 1:**
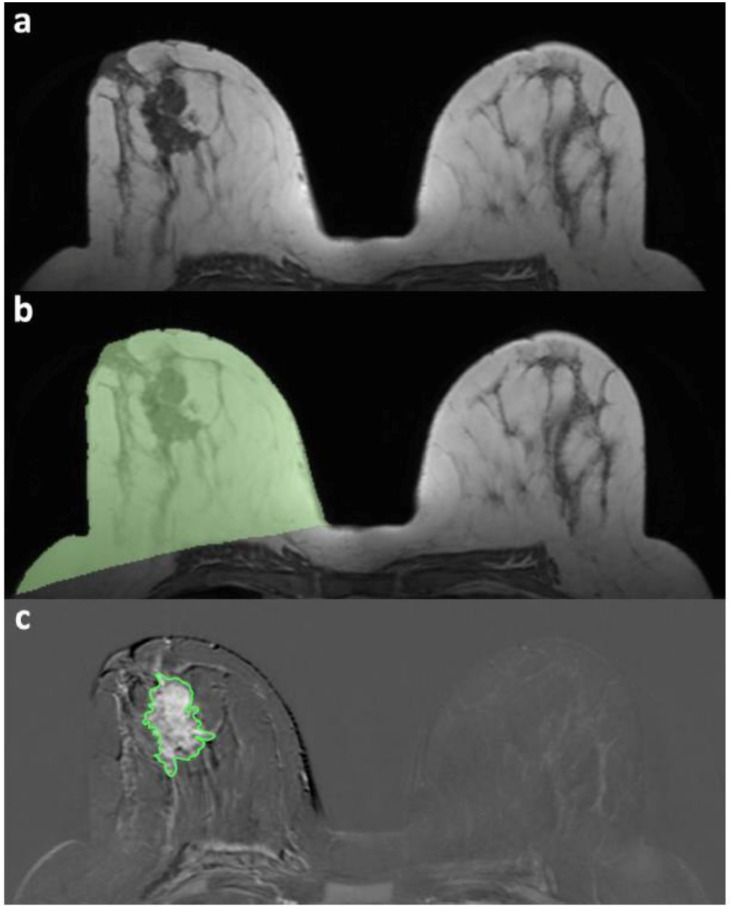
Example of a segmentation procedure for breast magnetic resonance volume metrics. (**a**) Definition of the slice with the largest breast area. (**b**) Whole breast volume segmentation with the region-growing method (green area); (**c**) fully manual segmentation of the tumour volume (green-contoured area).

After detailed instruction by a fellowship-trained breast radiologist with 12 years of breast MRI experience, two radiology residents, each of them with 2 years of breast MRI experience, were tasked with the segmentation of breast MRI images (Figure 1) using an open source Digital Imaging and Communications in Medicine (DICOM) viewer and processor (OsiriX Lite v.11.0, Pixmeo SARL, Bernex, Switzerland [27]). After a consensus reading of five cases, they independently reviewed all of the remaining cases according to the following task list, with access to pathology reports indicating the lesion localization in the affected breast.

First, the volume of the affected breast was segmented on an unenhanced T1-weighted series. For the overall breast volume assessment, the slice with the largest breast area was selected by measurement with an electronic calliper of the distance between the pectoralis muscles line and the breast–air anterior interface. The operator was asked to draw a region of interest that was expanded by the software on the superior and inferior slices by the threshold-wise inclusion of neighbouring voxels with a similar intensity. Second, a fully-manual segmentation of the tumour volume was performed on the subtracted dynamic contrast-enhanced series exhibiting the highest contrast enhancement, with the operator drawing a region of interest on each slice in which the lesion of interest was visible. The individual segmentation times for each task were automatically recorded. The TBVR was calculated as the per mille ratio (‰) between the tumour volume and breast volume.

### 2.5. Statistical Analysis

The Shapiro–Wilk test was used to determine the data distribution normality, after which the data were reported as mean ± standard deviation (SD) or median and interquartile range (IQR), according to their distribution being normal or non-normal, respectively. The tumour measurements at the final pathology, MRI-derived breast volume, tumour volume, TBVR, and segmentation times for each reader in each method were compared and correlated with the Wilcoxon test and Spearman’s ρ, used and interpreted as appropriate [28]. The Mann-Whitney *U* test was instead used to compare the TBVR between patients who underwent mastectomy and patients who underwent breast-conserving surgery.

In order to evaluate the interobserver reproducibility of the aforementioned MRI-derived metrics, we applied the Bland–Altman method [29], with the coefficients of repeatability (CoR) being calculated as 1.96 × SD of the differences of the two datasets. The reproducibility was reported as a complement to 100% of the ratio between the CoR and the mean. The bias (the mean of the differences between the two datasets) and 95% limits of agreement (bias ± 2 SD) were plotted as well. Statistical analyses were performed using SPSS v.26.0 (IBM SPSS Inc., Chicago, IL, USA), and statistical significance was set at *p* values < 0.05 [30].

## 3. Results

We retrieved, from our institutional database, a total of 170 women who underwent preoperative breast MRI following a biopsy-proven diagnosis of malignancy between 1 January 2013 and 31 May 2019. A total of 56 women had a unifocal mass lesion: the 5/56 (9%) women for whom surgery-related data was not available were analysed in consensus by the two readers as a training set. The remaining 51/56 women (median age 57 years, IQR 46.5–65.5 years) who complied to all of the other inclusion criteria constituted the study population (Table 2). Breast-conserving surgery was the surgical choice in 31/51 (61%) of the cases, while the remaining 20/51 (39%) underwent mastectomy of the affected breast. The semiautomatic segmentation of the whole breast volume and manual tumour volume segmentation was feasible in all of the included patients.

The segmentation by Reader 1 resulted in a median breast volume of 794 cm^3^ (IQR 607–1003 cm^3^), a median tumour volume of 1.43 cm^3^ (IQR 0.50–5.02 cm^3^), and a resulting median TBVR of 2.08‰ (IQR 0.70‰–9.13‰). Conversely, the segmentation by Reader 2 resulted in a median breast volume of 863 cm^3^ (IQR 600–1022 cm^3^), a median tumour volume of 1.76 cm^3^ (IQR 0.52–5.29 cm^3^), and a median TBVR of 2.28‰ (IQR 0.71‰–9.61‰). Table 3 details further characteristics of the two patient groups. Strong correlations with the final pathology tumour measurements (available in 41 cases) were found for the tumour volume segmentation by both readers (Reader 1 ρ = 0.748, *p* < 0.001; Reader 2 ρ = 0.778, *p* < 0.001), while a moderate to strong correlation was found for the TBVR obtained by both readers (Reader 1 ρ = 0.694, *p* < 0.001; Reader 2 ρ = 0.714, *p* < 0.001).

**Figure 2:**
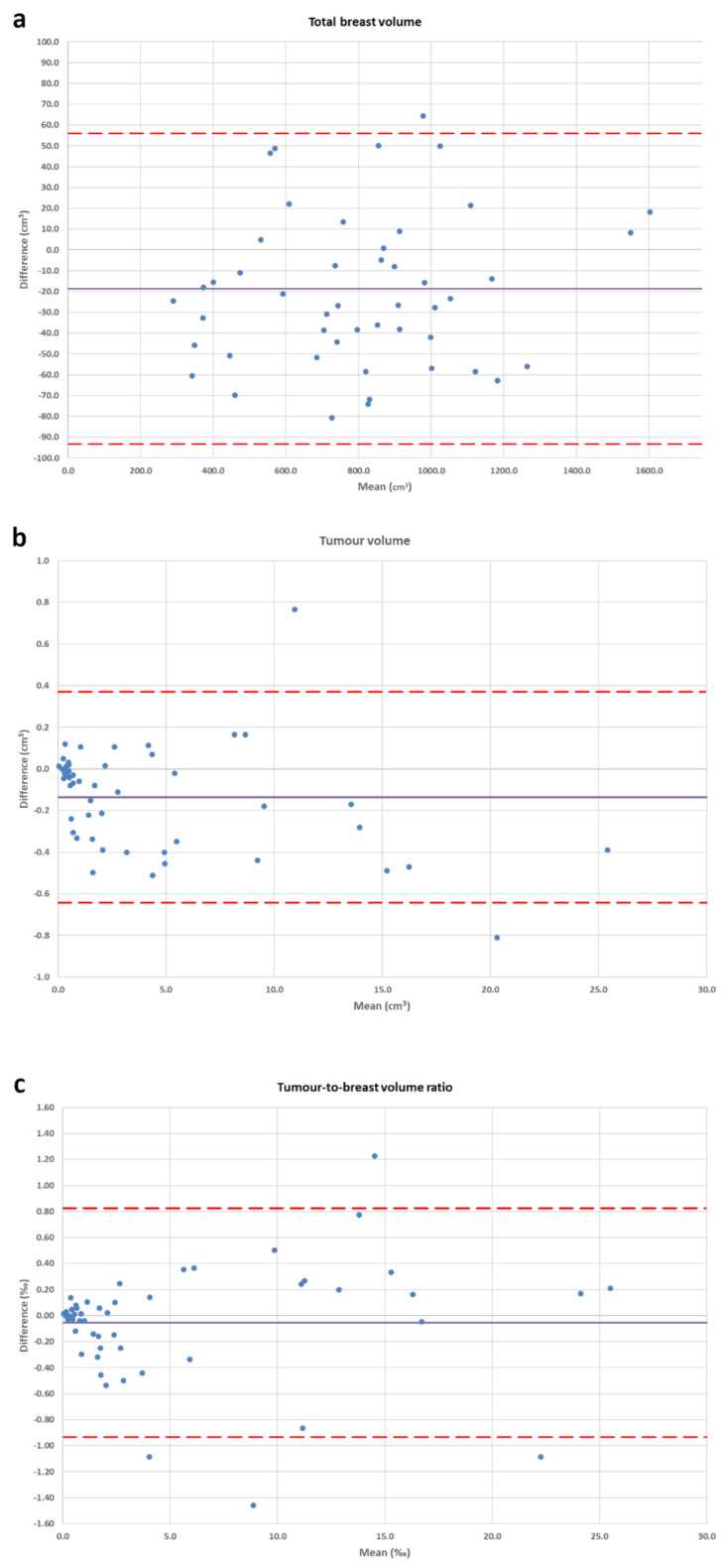
Bland–Altman plots for interobserver reproducibility. The straight and dashed lines represent the bias and its confidence interval, respectively. Blue dots represent the cases. (**a**) Whole breast volume region-growing segmentation; (**b**) fully manual segmentation of the tumour volume; (**c**) tumour-to-breast volume ratio.

The interobserver Bland–Altman analysis for the breast volume segmentation showed a CoR of 75 cm^3^ over a bias of −19 cm^3^, corresponding to 91% reproducibility; for the manual tumour volume segmentation, we found a 0.51 cm^3^ CoR over a bias of −0.14 cm^3^, corresponding to 88% reproducibility; for TBVR, we obtained a CoR of 0.88 over a 0.05 bias, corresponding to 84% reproducibility. The Bland–Altman plots are shown in Figure 2.

Reader 1’s segmentation times were 38 s (IQR 31–44 s) for the region-growing whole breast volume segmentation, and 129 s (IQR 91–191 s) for the manual tumour volume segmentation. Reader 2’s segmentation times were 69 s (IQR 54–82 s) for the region-growing whole breast volume segmentation, and 153 s (IQR 95–293 s) for the manual tumour volume segmentation.

**Figure 3:**
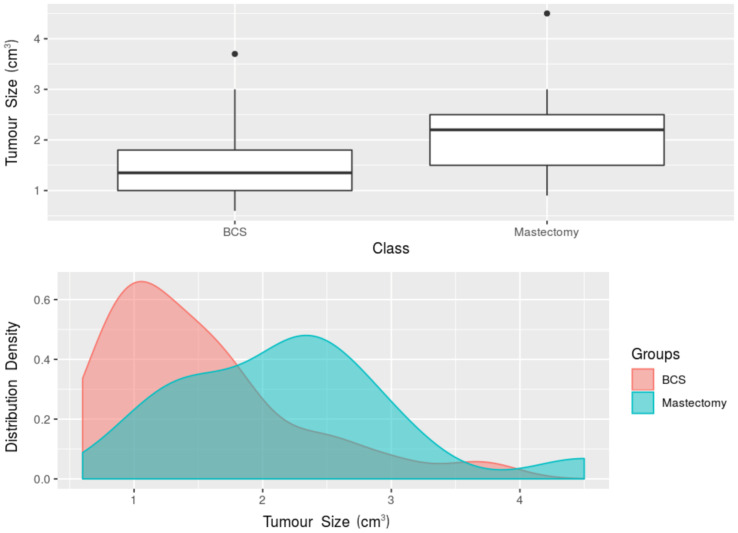
Boxplot and density plot for the comparison of tumour size at the final pathology between the women undergoing breast-conserving surgery and the women undergoing mastectomy. BCS, breast-conserving surgery.

**Figure 4:**
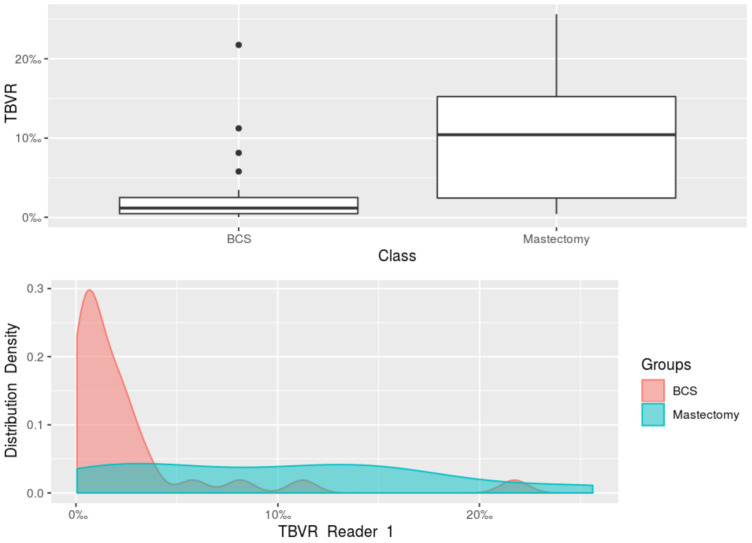
Boxplot and density plot for the comparison of the tumour-to-breast volume ratio obtained from Reader 1’s segmentations of MRI images of the women undergoing breast-conserving surgery and of the women undergoing mastectomy. BCS, breast-conserving surgery; TBVR, tumour-to-breast volume ratio.

**Figure 5:**
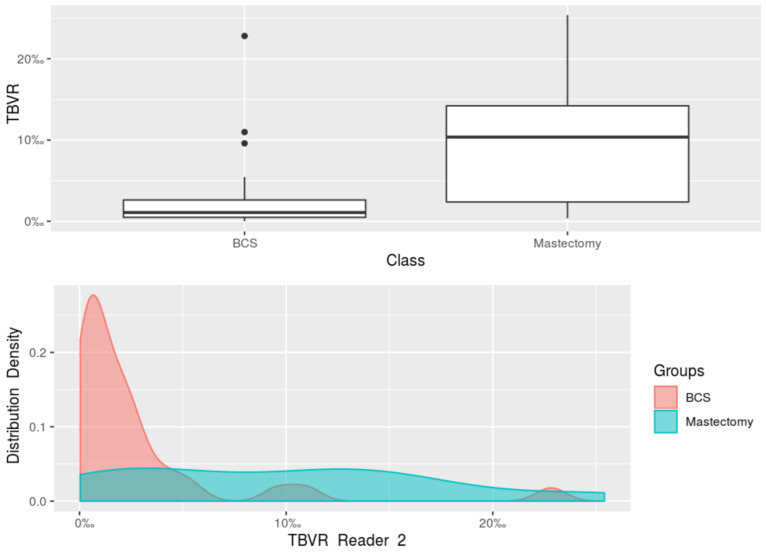
Boxplot and density plot for the comparison of the tumour-to-breast volume ratio obtained from Reader 2’s segmentations of MRI images of the women undergoing breast-conserving surgery and of the women undergoing mastectomy. BCS, breast-conserving surgery; TBVR, tumour-to-breast volume ratio.

When the women who underwent breast-conserving surgery and those who underwent mastectomy were compared, the Mann-Whitney *U* test found a statistically significant difference (*p* = 0.008) between the median tumour size at the final pathology (available for 41 women), with a median 2.20 cm tumour size (IQR 1.50–2.50 cm) in the women who underwent mastectomy, and a median 1.35 cm tumour size (IQR 1.00–1.80 cm) in the women who underwent breast-conserving surgery (Figure 3). This difference was mirrored by the TBVR values: overall, lower TBVR values (*p* < 0.001) were observed in the breast-conserving surgery group (median 1.14‰, interquartile range 0.49–2.55‰) than in the mastectomy group (median 10.52‰, interquartile range 2.42–14.73‰). Also considering reader-specific assessment, Reader 1’s measurements showed a median TBVR of 10.43‰ (IQR 2.46–15.23‰) in the women who underwent mastectomy, and a median TBVR of 1.19‰ (IQR 0.49–2.51‰) in the women who underwent breast-conserving surgery (*p* < 0.001, Figure 4). Reader 2’s measurements yielded a median TBVR of 10.37‰ (IQR 2.39–14.23‰) in the women who underwent mastectomy, and a median TBVR of 1.09‰ (IQR 0.49–2.64‰) in the women who underwent breast-conserving surgery (*p* < 0.001, Figure 5).

## 4. Discussion

For early-stage invasive breast cancer the standard of care is breast-conserving surgery followed by whole-breast radiation therapy [31]. While improving overall cosmetic results compared to mastectomy (nonetheless achieving acceptable surgical outcomes), breast-conserving surgery can still lead to postoperative aesthetic deformity or involved margins [9]. TBVR, as measured by the preoperative MRI, is an objective parameter which could contribute to the surgical decision of whether to perform breast-conserving surgery or a mastectomy [20,21,22], while also being potentially applied as a predictive factor of the cosmetic outcome in breast cancer patients undergoing conserving surgery [16,17,23]. The purpose of this study was to investigate and address several factors which are perceived as limiting TBVR’s wider introduction into clinical practice, such as the fact that manual MRI image segmentation is time-consuming and potentially heavily operator-dependent [20]. We chose to focus initially on 51 biopsy-proven B5 lesions with a mass-like appearance on MRI, as the easiest category to reproducibly locate (and segment) on MRI images, tasking two radiology residents with the image processing after dedicated instruction. Our choice to use open-source software aimed to maximize the external reproducibility of our study, whilst also testing the hypothesis that an embedded semi-automatic segmentation method (the so-called ‘region-growing technique’) for the whole breast volume segmentation could sizably curtail the segmentation times without compromising the interobserver reproducibility. As expected, the manual tumour volume segmentation yielded both high interobserver reproducibility (88%) and a strong correlation with the pathology (ρ ≥ 0.748), with the semiautomatic region-growing segmentation achieving a similarly high interobserver reproducibility (91%), with segmentation times between 60% and 40% lower than manual tumour volume segmentation. The ensuing total segmentation time to obtain the TBVR would therefore be about 3–4 min, which is suitable for integration into clinical practice.

The moderate to strong correlations (ρ ≥ 0.694) found between the TBVR obtained by both readers and tumour size at final pathology highlight the immediate correspondence between this index and pathological correlates, potentially confirming its clinical reliability. However, the absence of an even stronger correlation could be explained in favour of the TBVR by considering the way in which this index can easily take into consideration the whole tumour’s three-dimensional volume, while standard pathological measurements on a surgical specimen are based on two-dimensional diameters measured on a single cut of the surgical specimen [32].

In the comparison between the two treatment groups of this study, i.e., the 31 women (61%) who received breast-conserving surgery and the 20 women (39%) who underwent mastectomy, we found—as expected [9]—a significant difference between the tumour sizes at the final pathology (*p* = 0.008), mirrored however by an even more polarized difference in their TBVR values (*p* < 0.001), which were almost ten times lower in the breast-conserving surgery group for both readers; this is an even more pronounced difference than those reported by the only three other studies that have—up to now—investigated this topic [20,22,24].

Further multicentric prospective studies are warranted in order to foster the integration of TBVR into clinical practice, chiefly by precisely defining the TBVR classes and cut-offs correlated with surgical choices. Hypothesizing five TBVR classes, the two extremes (classes 1 and 5) would comprise TBVR values in which mastectomy and breast-conserving surgery are almost invariably performed. Classes 2 and 4 would represent an intermediate-to-high probability of receiving each surgery, with limited discretional variations justified by recognized factors, such as a pre-existing high risk of recurrence (e.g., in BRCA positive patients) [33,34] or patient preferences [33], or by tumour-specific features such as multifocality [33,34], location in quadrants that are at higher risk for postoperative breast deformity [11], wide extension preventing the obtainment of an optimal margin width and increasing re-excision risks [35,36,37,38]. These factors would even more deeply interplay with TBVR in the middle class, 3, in which multidisciplinary evaluation would still be the main determinant of the surgical choices. The potential integration of TBVR into a clinical routine would also offer to surgeons—and to all of the other specialists involved in the tumour board—quantitative information to facilitate the discussion and overcome subjective considerations based on rough estimations of breast size according to cups. The need for such a metric, while still underrepresented in the literature [13,22,23], has been acknowledged by the most recent guidelines issued by the European Society of Medical Oncology [39], which recommends the consideration of tumour size in relation to breast size, both in the choice between mastectomy and breast-conserving surgery and in the planning of oncoplastic surgery. Moreover, TBVR would represent a readily intelligible parameter to be discussed with patients, fostering higher patient awareness in surgical planning and favouring an easier understanding of cosmetic results.

The limitations of our study, other than its retrospective nature and limited sample size, include the limited experience of the two readers; however, the fairly high reproducibility obtained for all of the investigated metrics, and the reduced total segmentation times scored by the relatively unexperienced readers, highlight the way in which such indexes could be even more smoothly introduced into clinical practice by dedicated radiologists. Another double-sided limitation involves the exclusive focus on mass-like lesions at breast MRI, and the relatively small median size of the analysed lesion, which were all detected in an organized or opportunistic screening. Another limitation linked to this last one was the impossibility of conducting the semiautomatic region-growing segmentation on multifocal tumours with the selected software. Of note, we exploratively applied region-growing segmentation to the subset of 32 unifocal lesions (see Tables S1 and S2, Figures S1 and S2) with a sizable reduction in their segmentation times, a strong correlation with their final pathology measurements, and a high tumour volume and TBVR reproducibility. In this regard, the application of artificial intelligence—and particularly of deep learning—for fully automatic image segmentation in multifocal lesions could improve both the accessibility and the intrinsic fit of these indexes [40].

In conclusion, our study showed that TBVR and its related metrics can be obtained from a breast MRI with good to high reproducibility—even by relatively unexperienced readers—in about 4 min, whilst also demonstrating the strong direct relationship between TBVR and subsequent surgical planning.

## Figures and Tables

**Table 1:** Breast magnetic resonance technical parameters for each acquired sequence.

Sequence	TR (ms)	TE (ms)	Flip Angle	*b* Values (s/mm^2^)	Spatial Resolution (mm)	Matrix (Voxels)	Slices	Field of View (mm^3^)	Time (mm:ss)
**STIR**	9670	75.00	150°	-	0.7 × 0.7 × 2.0	512 × 512	60	358.4 × 358.4 × 120	08:24
**DWI**	4900	76.00	90°	0, 750	2.7 × 2.7 × 4.0	60 × 172	30	162 × 464.4 × 120	03:31
**DCE-MRI**	11	4.89	45°	-	0.8 × 0.8 × 1.3	512 × 512	120	409.6 × 409.6 × 156	09:55

TR: Repetition time; TE: Echo time; STIR: Short-tau inversion recovery; DWI: Diffusion weighted imaging; DCE-MRI: Dynamic contrast-enhanced magnetic resonance imaging.

**Table 2:** Demographics, staging, and pathology findings.

Variables		Breast-Conserving Surgery (31 Patients)	Mastectomy (20 Patients)
Median age (IQR)		59.0 years (47.5–68.0 years)	52.5 years (45.0–59.7 years)
**MRI staging**			
T1a (%)		1 (3%)	-
T1b (%)		7 (23%)	2 (10%)
T1c (%)		14 (45%)	4 (20%)
T2 (%)		8 (26%)	12 (60%)
T3 (%)		-	2 (10%)
T4b (%)		1 (3%)	-
Final pathology *			
Median tumour size at final pathology (IQR)		1.35 cm (1.00–1.80 cm)	2.20 cm (1.50–2.50 cm)
**Pathology diagnosis**	IDC (%)	6 (21%)	10 (50%)
	DCIS (%)	-	1 (5%)
	ILC (%)	-	3 (15%)
	IDC + DCIS (%)	16 (55%)	5 (25%)
	ILC + LCIS (%)	6 (21%)	1 (5%)
	Micropapillary carcinoma (%)	1 (3%)	-
Molecular subtypes **	Luminal A type (%)	7 (24%)	5 (28%)
	Luminal B type (%)	19 (66%)	9 (50%)
	HER2 overexpression type (%)	1 (3%)	1 (5%)
	Triple negative type (%)	2 (7%)	3 (17%)

* Final pathology was not available for two patients. ** Molecular subtypes were not available for four patients. IQR, interquartile range; MRI, magnetic resonance imaging; IDC, invasive ductal carcinoma; DCIS, ductal carcinoma in situ; LCIS, lobular carcinoma in situ; ILC, invasive lobular carcinoma.

**Table 3:** Breast magnetic resonance volume metrics and segmentation times across the patient groups.

Variables		Breast-Conserving Surgery (31 Patients)	Mastectomy (20 Patients)
**Breast MRI Volume Metrics**	Median breast volume Reader 1 (IQR)	789.05 cm^3^ (587.12–1022.83 cm^3^)	837.43 cm^3^ (687.38–982.93 cm^3^)
	Median breast volume Reader 2 (IQR)	863.20 cm^3^ (539.50–1022.10 cm^3^)	857.54 cm^3^ (723.52–999.46 cm^3^)
	Median tumour volume Reader 1 (IQR)	0.69 cm^3^ (0.40–1.91 cm^3^)	6.81 cm^3^ (2.00–11.95 cm^3^)
	Median tumour volume Reader 2 (IQR)	0.85 cm^3^ (0.39–2.21 cm^3^)	6.74 cm^3^ (2.07–11.45 cm^3^)
	Median TBVR Reader 1 (IQR)	1.19‰ (0.49–2.51‰)	10.43‰ (2.46–15.23‰)
	Median TBVR Reader 2 (IQR)	1.09‰ (0.49–2.64‰)	10.37‰ (2.39–14.23‰)
**Segmentation Times**	Breast volume Reader 1 (IQR)	38 s (31–44 s)	
	Breast volume Reader 2 (IQR)	69 s (54–82 s)	
	Tumour volume Reader 1 (IQR)	129 s (91–191 s)	
	Tumour volume Reader 2 (IQR)	153 s (95–293 s)	

IQR, interquartile range; MRI, magnetic resonance imaging; TBVR, tumour-to-breast volume ratio.

## Data Availability

All data analysed for this study are presented in the manuscript or in the supplementary material.

## Supplementary Materials

The following are available online at https://www.mdpi.com/2075-4418/11/2/204/s1, Table S1: Comparison of the volume metrics obtained by applying both manual segmentation and region-growing segmentation to the subset of 32 patients with 32 unifocal lesions; Table S2: Comparison of the segmentation times obtained by applying both manual segmentation and region-growing segmentation to the subset of 32 patients with 32 unifocal lesions; Figure S1: Bland–Altman plot for the interobserver reproducibility of the region-growing segmentation of tumour volume; Figure S2: Bland–Altman plot for the interobserver reproducibility of the tumour-to-breast volume ratio obtained by the region-growing segmentation of the tumour volume.
