# Supplementary material for: MRI-Derived Tumour-to-Breast Volume Is Associated with the Extent of Breast Surgery
Source: Diagnostics (Basel). 2021 Jan 30;11(2):204. doi: 10.3390/diagnostics11020204 (PMC7912531; doi:10.3390/diagnostics11020204)
Supplement: Supplementary file 1 [file diagnostics-11-00204-s001.pdf]

diagnostics

MDPI

Table S1. Comparison of volume metrics obtained by applying both manual segmentation and region growing segmentation to the subset of 32 patients with 32 unifocal lesions.

	Patients who Underwent Breast-Conserving Surgery (23 patients)	Spearman's ϱ	Patients who Underwent Mastectomy (9 patients)	Spearman's ϱ
Median MS-TBVR Reader 1 (IQR)	1.19‰ (0.49‰–2.57‰)	0.986	4.11‰ (1.34‰–6.29‰)	0.983
Median RG-TBVR Reader 1 (IQR)	1.08‰ (0.51‰–2.60‰)		3.42‰ (1.20‰–6.55‰)	
Median MS-TBVR Reader 2 (IQR)	1.09‰ (0.49‰–2.94‰)	0.984	3.97‰ (1.49‰–6.08‰)	0.983
Median RG-TBVR Reader 2 (IQR)	1.02‰ (0.50‰–2.40‰)		2.86‰ (1.11‰–6.03‰)	
Median MS-tumour volume Reader 1 (IQR)	0.70 cm^3 (0.40–1.91 cm^3)	0.980	2.71 cm^3 (0.52–4.38 cm^3)	0.967
Median RG-tumour volume Reader 1 (IQR)	0.74 cm^3 (0.31–1.67 cm^3)		2.26 cm^3 (0.50–4.58 cm^3)	
Median MS-tumour volume Reader 2 (IQR)	0.99 cm^3 (0.39–2.21 cm^3)	0.971	2.82 cm^3 (0.74–4.64 cm^3)	0.983
Median RG-tumour volume Reader 2 (IQR)	0.74 cm^3 (0.33–1.57 cm^3)		2.04 cm^3 (0.64–4.39 cm^3)	

IQR, interquartile range; MS, manual segmentation; RG, region growing segmentation; TBVR, tumour-to-breast volume ratio.

Table S2. Comparison of segmentation times obtained by applying both manual segmentation and region growing segmentation to the subset of 32 patients with 32 unifocal lesions.

Tumour Volume Segmentation Time	Reader 1	Reader 2	Wilcoxon Test *p* Value
Manual method (IQR)	124 s (86–158 s)	138 s (92–198 s)	0.905
Region growing method (IQR)	33 s (25–46 s)	32 s (25–49 s)	0.999
**Wilcoxon test *p* value**	< 0.001	< 0.001	

IQR, interquartile range.

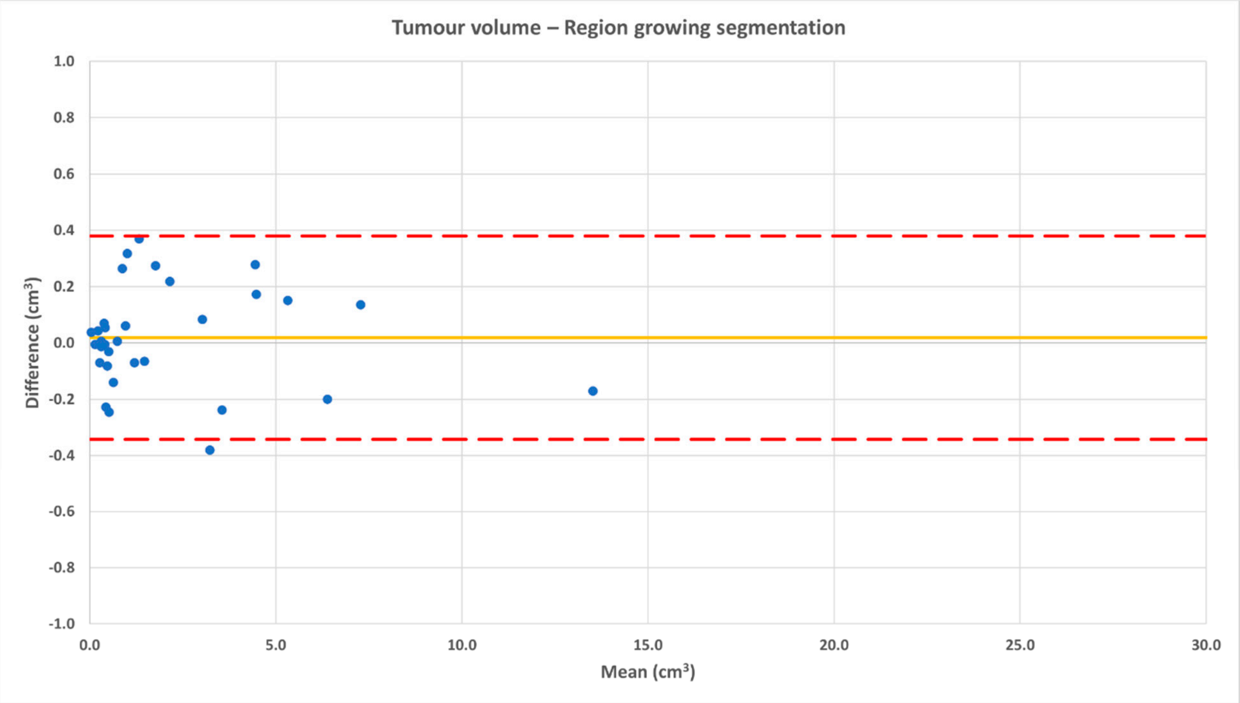

Figure S1. Bland–Altman plot for interobserver reproducibility of region growing segmentation of tumour volume. Coefficient of reproducibility: 0.36 cm^3. Bias: 0.02 cm^3 bias. Reproducibility: 83%.

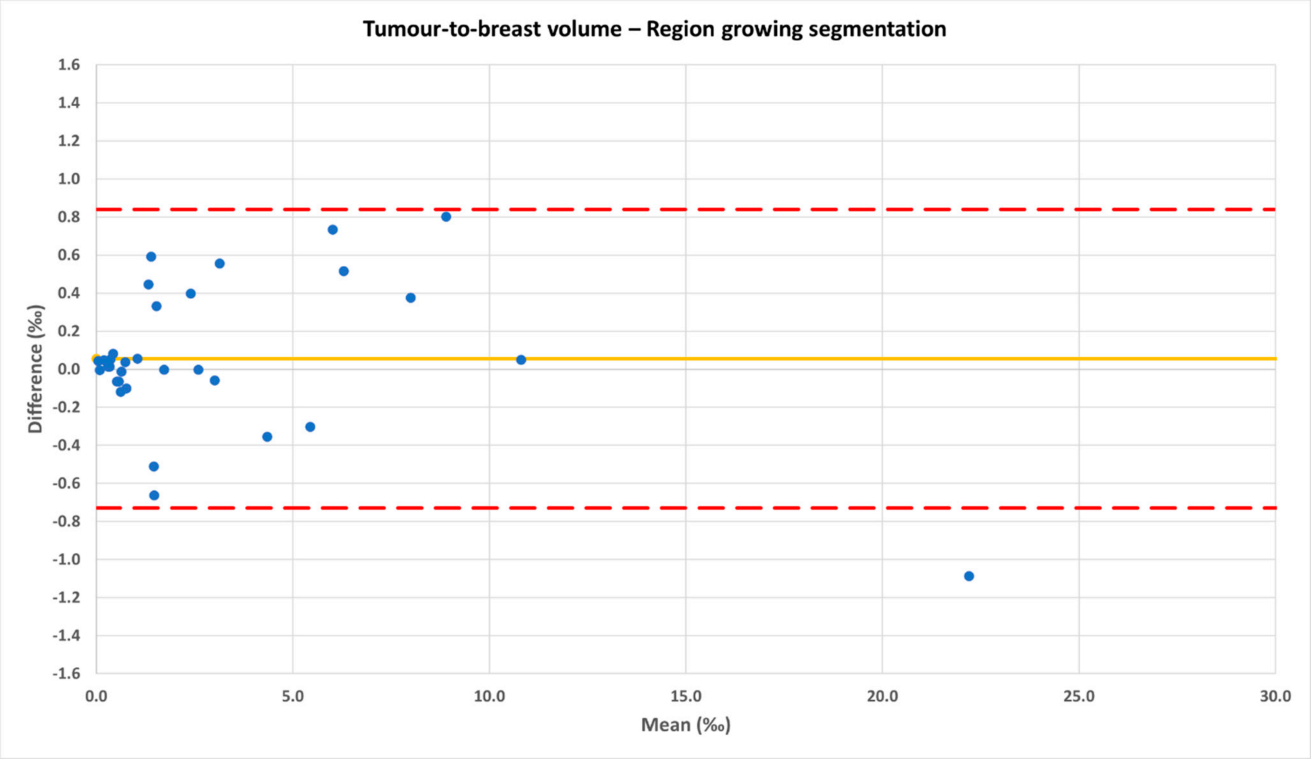

Figure S2. Bland–Altman plot for interobserver reproducibility of tumour-to-breast volume ratio obtained with region growing segmentation of tumour volume. Coefficient of reproducibility: 0.78 cm^3. Bias: 0.06 cm^3 bias. Reproducibility: 75%.
